# Global Marine N_2_ Fixation Estimates: From Observations to Models

**DOI:** 10.3389/fmicb.2018.02112

**Published:** 2018-09-19

**Authors:** Angela Landolfi, Paul Kähler, Wolfgang Koeve, Andreas Oschlies

**Affiliations:** GEOMAR Helmholtz Centre for Ocean Research Kiel, Kiel, Germany

**Keywords:** marine N_2_ fixation, N-cycle balance, denitrification, marine productivity, marine heterotrophic diazotrophy, nitrogen isotopes

## Abstract

Fixed nitrogen (N) limits productivity across much of the low-latitude ocean. The magnitude of its inventory results from the balance of N input and N loss, the latter largely occurring in regionally well-defined low-oxygen waters and sediments (denitrification and anammox). The rate and distribution of N input by biotic N_2_ fixation, the dominant N source, is not well known. Here we compile N_2_ fixation estimates from experimental measurements, tracer-based geochemical and modeling approaches, and discuss their limitations and uncertainties. The lack of adequate experimental data coverage and the insufficient understanding of the controls of marine N_2_ fixation result in high uncertainties, which make the assessment of the current N-balance a challenge. We suggest that a more comprehensive understanding of the environmental and ecological interaction of marine N_2_ fixers is required to advance the field toward robust N_2_ fixation rates estimates and predictions.

## Introduction

Marine N_2_ fixation, the largest source of fixed N to the ocean, maintains ocean fertility by compensating for N-losses via denitrification. Global warming, likely inducing ocean deoxygenation (Keeling et al., 2010), and increasing N load from the atmosphere and/or rivers, are perturbing the N cycle leading to increasing N loss via denitrification on the one hand (Codispoti et al., 2001; Codispoti, 2007) and to alterations of the niche of marine N_2_ fixers on the other (Landolfi et al., 2017), with an unknown net effect on the N inventory. A prolonged mismatch between N inputs and losses would cause changes to the oceanic fixed N inventory, potentially affecting ocean productivity and ocean carbon (C) storage. Narrowing down the uncertainties in current N_2_ fixation estimates is key to diagnosing any imbalance in the marine N budget and its effect on the marine C budget.

N_2_ fixation can be inferred experimentally from incubation assays (Capone, 1993; Montoya et al., 1996), and from its geochemical imprint on nutrient (Gruber and Sarmiento, 1997) and stable N isotope distributions (Altabet, 2007). Geochemical estimates use the integrated N_2_ fixation signature over large scales of space and time, smoothing any small-scale variability inherent in experimental N_2_ fixation measurements. Traditionally, geochemical (Gruber, 2004) and experimental (Capone et al., 2005) estimates suggested highest N_2_ fixation rates in the Tropical North Atlantic, remote from the region of largest pelagic N-loss in the Eastern Tropical Pacific. Although such spatial separation was difficult to reconcile with paleo-oceanographic evidence of a closely balanced N-cycle, considered to require tight stabilizing feedbacks between N-loss and N_2_ fixation (Altabet, 2007), it was in line with the well-documented high energy and iron requirements of diazotrophs (Kustka et al., 2003) that would restrict them to warm and iron-rich waters. This view has been challenged by a model-based geochemical N_2_ fixation estimate suggesting prevailing control by N deficits and close spatial coupling of N_2_ fixation and N loss (Deutsch et al., 2007), and by new evidence from molecular techniques demonstrating a wider diversity of N_2_ fixers and N_2_ fixation strategies. This includes free-living unicellular cyanobacteria (Zehr et al., 1998; Montoya et al., 2004), cyanobacterial symbionts and heterotrophic phylotypes (see Foster et al., 2011; Bombar et al., 2016; Caputo et al., 2018), covering novel habitats such as aphotic waters (Bonnet et al., 2013; Benavides et al., 2015, 2016, 2018) and oxygen deficient zones (ODZs) (Fernandez et al., 2011; Jayakumar et al., 2012; Bonnet et al., 2013; Löscher et al., 2014, 2016; Turk-Kubo et al., 2014; Knapp et al., 2016). Recently, severe problems in applying the N_2_ fixation assays came to light (Mohr et al., 2010). Considering this new evidence, using a constant correction factor of historical data, a doubling of N_2_ fixation has been suggested (177 ± 8 Tg N y^−1^, Großkopf et al., 2012). Despite the pace of new discoveries, global N_2_ fixation estimates remain highly uncertain (Gruber, 2016). The incomplete knowledge of the organisms involved and their physiological and ecological controls prevent any robust prediction of their distribution and activity. Here we present a brief overview of recent work covering experimental, geochemical, and model-based N_2_ fixation estimates and discuss knowledge gaps. We suggest future research strategies to reduce the current uncertainty.

## Experimental Estimates

**FIGURE 1:**
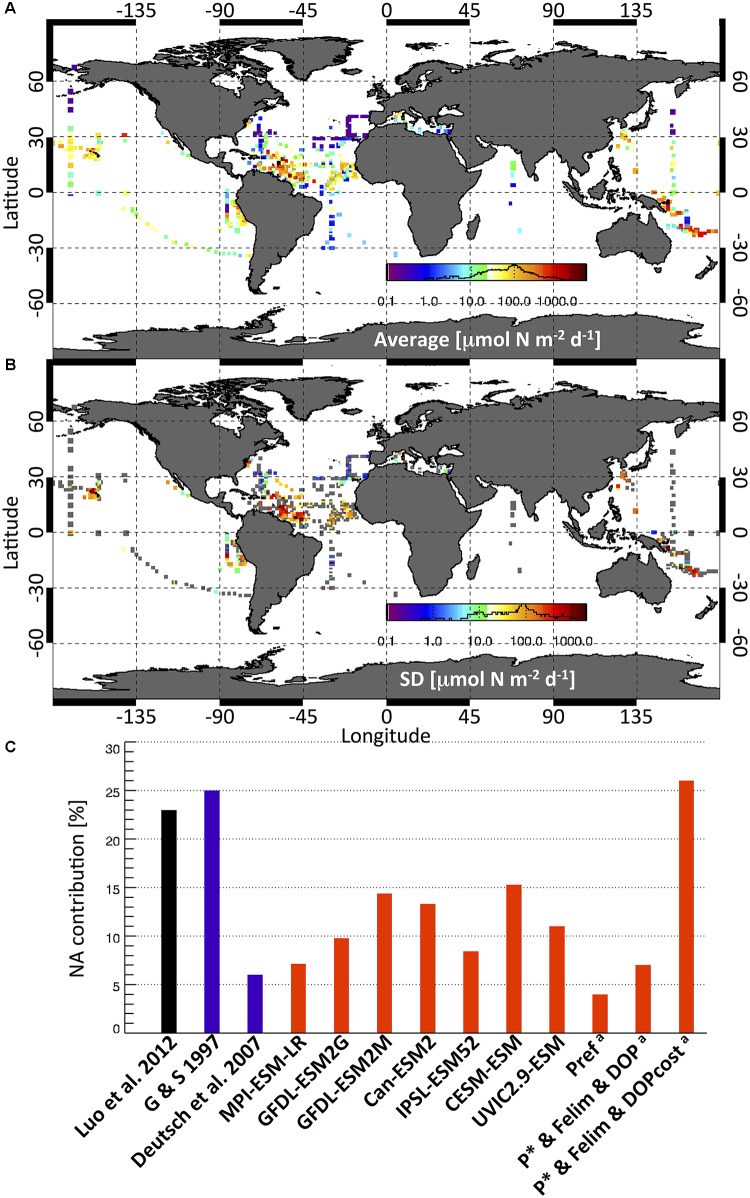
1 × 1 degree grid **(A)** average and **(B)** standard deviation (SD) of depth integrated N_2_ fixation rate (μmol N m^−2^ d^−1^) experimental measurements from the MAREDAT database (Luo et al., 2012) and 800 additional data points (see text for details). Gray squares in SD plot indicate one-only data point. Data distribution histogram is shown in the color bar. **(C)** Experimental (black)-, geochemical (blue)-, and model (red) -based estimated contribution (%) of the North Atlantic to global N_2_ fixation rates. Models include those of the Coupled Model Intercomparison Project (CMIP5, see **Table 1**), a model with additional energetic costs of DOP uptake (UVIC2.9-ESM, Landolfi et al., 2017) and ^a^model experiments of Landolfi et al. (2015) accounting for preferential phosphorus remineralization (PREF), P^∗^ and iron limitation and DOP uptake without (P^∗^ and Fe and DOP), and with additional energetic and also N costs (P^∗^ and Fe and DOPcost).

The compilation of surface marine N_2_ fixation rate measurements (MARine Ecosystem DATa) by Luo et al. (2012), with more recent data from the North Pacific (Shiozaki et al., 2015a,b, 2017), western Pacific (Bonnet et al., 2009, 2015, 2017, 2018; Shiozaki et al., 2013, 2014b; Berthelot et al., 2017), eastern tropical South Pacific (Löscher et al., 2014, 2016; Knapp et al., 2016), Indian Ocean (Shiozaki et al., 2014a), and the tropical Atlantic (Großkopf et al., 2012; Singh et al., 2017), is presented in **Figure 1**. The measurements reported are mostly from bulk unfiltered upper ocean samples, including cyanobacteria and potentially other diazotrophs. Although highly variable, the highest depth-integrated N_2_ fixation rates are found in the western tropical South Pacific (638 ± 1689 μmol N m^−2^ d^−1^, 201 profiles). These are larger than the traditionally high rates in the subtropical North Atlantic (182 ± 479 μmol N m^−2^ d^−1^, 636 profiles) and North Pacific (118 ± 101 μmol N m^−2^ d^−1^, 272 profiles). In the phosphate-rich waters of the eastern South Pacific average N_2_ fixation is 86 ± 99 μmol N m^−2^ d^−1^ (213 profiles). Low rates are found in the southern Indian Ocean (<20 μmol N m^−2^ d^−1^, Shiozaki et al., 2014a), and in cold Bering Sea waters (10 μmol N m^−2^ d^−1^, Shiozaki et al., 2017). Based on the MAREDAT database, Luo et al. (2012) estimated a global N_2_ fixation rate of 137 ± 9.2 Tg N y^−1^ (**Table 1**). Global extrapolations are difficult as observations remain sparse and highly variable in space and time (**Figure 1**). Only few ocean regions have sufficient data coverage to assess spatial, seasonal, or inter-annual variability (SD, **Figure 1B**). The large standard deviation in the western tropical South Pacific suggests a large spatio-temporal variability. The North Atlantic variability appears related to the strong spatial and temporal gradients of physical forcing and associated environmental factors (e.g., Mouriño-Carballido et al., 2011; Landolfi et al., 2016). The variability in the North Pacific is mostly associated with seasonal changes (Böttjer et al., 2016) and variable mesoscale activity (e.g., Church et al., 2009).

**Table 1:** Experimental, geochemical, and ESM-based (1996–2005 average) regional and global annual N_2_ fixation estimates (Tg N y^−1^).

	Pacific	Indian	Atlantic	Global
Source	Tg N y^−1^	Tg N y^−1^	Tg N y^−1^	Tg N y^−1^
Luo et al., 2012	102 ± 20	–	34 ± 7	137 ± 9
Gruber and Sarmiento, l997			28	110 ± 40
Deutsch et al., 2007^1^	95	22	20	137
CMIP5-MPI-ESM-LR^a^	132	32	38	213
CMIP5-GFDL-ESM2G^b^	106	28	36	181
CMIP5-GFDL-ESM2M^b^	75	30	40	154
CMIP5-CanESM2^c^	72	28	28	130
CMIP5-IPSL-CM52-LR^2,d^	54	14	16	89
CMIP5-CESM^e^	72	48	46	173
ESM-UVIC2.9^f^	73	31	24	128

*^1^Estimate from the Standard model that includes DOP^∗^, which is 5% higher than P^∗^-only estimate. Coupled Model Intercomparison Project (CMIP5) model output has been retrieved from https://esgf-node.llnl.gov/projects/cmip5/. ^2^Estimated from model C:N = 122:16. References a–f below list the model descriptions of the respective biogeochemical modules. ^a^Ilyina et al., 2013; ^b^Dunne et al., 2013; ^c^Zahariev et al., 2008; ^d^Aumont et al., 2015; ^e^Moore et al., 2013; and ^f^Landolfi et al., 2017.*

Experimental measurements have technical drawbacks. The acetylene reduction assay (Capone, 1993), a proxy measurement of both incorporated and exuded (gross) fixed N_2_, measures the rate of ethylene production from added acetylene. Microbial activity repression (Fulweiler et al., 2015), low sensitivity and highly variable conversion factors for C_2_H_4_:N_2_ (Wilson et al., 2012), limit the robustness of this technique. The more widely (∼75% of the measurements) applied method involves adding ^15^N_2_ gas to a water sample and measuring the net incorporation of ^15^N_2_ into plankton biomass after filtration (Montoya et al., 1996). This method may result in significant underestimation of N_2_ fixation rates if the added ^15^N_2_ gas is not equilibrated before the start of the incubation (Mohr et al., 2010; Wilson et al., 2012; Wannicke et al., 2018). The degree of underestimation is variable and dependent on the experimental conditions (incubation time, size of bottle, shaking vigor), making the correction of historical data with a constant factor arbitrary. The ^15^N_2_ gas stock contamination by ^15^NH_4_^+^ or ^15^NO_x_, may result in N_2_ fixation overestimate (Dabundo et al., 2014). Fixation rates may be underestimated if small cells (<2 μm) are lost during filtration, particularly affecting rates where small diazotrophs may dominate the N_2_-fixing community (Bombar et al., 2018). Robust quantification of low rates is currently limited by the low sensitivity and high potential errors of present methods (Gradoville et al., 2017; Moisander et al., 2017). Inadequate consideration of experimental biases must be warned against. Community efforts are being made toward a consensus on methods and protocols refinement^1^ for yielding more accurate and comparable fixation rates.

## Geochemical Estimates

Geochemical estimates of N_2_ fixation have been inferred from excess NO_3_ with respect to the Redfield-equivalent PO_4_ (the N^∗^ method), constructed from the high-quality global nutrient surveys (e.g., JGOFS/WOCE; Conkright et al., 2002), combined with information on ocean ventilation ages derived from measurements of abiotic transient tracers such as chlorofluorocarbons (CFCs). This method rests on the assumption that nutrients’ departure from the global average ratio of NO_3_:PO_4_ = 16:1 (RR, the Redfield ratio) is due to N_2_ fixation adding (N:P > RR), and denitrification removing (N:P < RR), nitrogen. This approach has been used to compute North Atlantic N_2_ fixation rates (Michaels et al., 1996; Gruber and Sarmiento, 1997; Hansell et al., 2004, 2007; Kähler et al., 2010), and derive a global estimate of 110 ± 40 Tg N y^−1^ (Gruber and Sarmiento, 1997; **Table 1**). While this method has the advantage of integrating over large scales of space and time and implicitly includes potential contributions of non-cyanobacterial N_2_ fixation, several potential shortcomings have been discussed: Impacts of non-Redfield remineralization of organic matter were addressed in Landolfi et al. (2008), where we extended the inorganic N^∗^ concept to total nitrogen excess, TN_xs_, to include organic nutrients. We found that for the subtropical North Atlantic, N_2_ fixation rates estimated via TN_xs_ were approximately twice as high as those estimated by the N^∗^ method, but cautioned that other processes such as atmospheric N deposition (Zamora et al., 2010) and non-Redfield uptake of nutrients by phytoplankton (Mills and Arrigo, 2010; Weber and Deutsch, 2012), can also affect regional patterns of oceanic N^∗^ and TN_xs_ and thereby influence N_2_ fixation rate estimates. These caveats also apply to the P_t_^∗^ method introduced by Deutsch et al. (2007) that estimates N_2_ fixation from the consumption of P_t_^∗^, which is defined as total excess P relative to the Redfield N equivalent. In contrast to the classical N^∗^ tracer, but similar to TN_xs_, P_t_^∗^ also accounts for organic nutrients. Deutsch et al. (2007) estimated a global N_2_ fixation rate of 137 Tg N y^−1^ (i.e., about 25% higher than the N^∗^-based estimate), with a very large contribution from surface waters in close proximity to the largest ODZ in the eastern South Pacific and only a small contribution from the P_t_^∗^-poor North Atlantic (**Table 1**). The P_t_^∗^-based estimate contrasts with the prevailing view of elevated N_2_ fixation rates in the North Atlantic, as indicated both from N^∗^ and experimental evidence (**Figure 1**). Besides the above mentioned non-Redfield processes other than N_2_ fixation errors in the rate estimates also arise from the assumed ocean circulation that generally is not fully consistent with the observed nutrient fields to which it is applied. While current-generation global circulation models represent global-scale patterns reasonably well, they have particular deficiencies in reproducing low-oxygen regions (Cabré et al., 2015) and tracer distributions in the tropical regions (Dietze and Löptien, 2013). The refinement of global ocean GCMs is an on-going process. However, improvements to the geochemical methods may be limited given that the available nutrient distributions represent composites of data from decad of observations from a turbulent ocean, for which no underlying “mean” circulation may exist. Errors may also stem from uncertainties in tracer-derived water ages and age-based rate estimates: The differential effects of mixing on water age versus nutrient tracers may lead to errors in tracer-based N_2_ fixation estimates (Koeve and Kähler, 2016).

## Model-Based Estimates

Global N_2_ fixation estimates have been derived from models using both implicit (diagnostic) and explicit (prognostic) N_2_ fixation parameterizations. Implicit N_2_ fixation parameterizations used in some IPCC-type earth-system-models (ESM) are based on restoring-type approaches that simulate N_2_ fixation by restoring upper-ocean nutrient ratios toward Redfield NO_3_:PO_4_ stoichiometry (MPI-ESM, Ilyina et al., 2013). This implies that N_2_ fixation is mainly restricted to areas with observed low NO_3_:PO_4_ ratios. Other models restrict N_2_ fixation to N limited areas, but account also for light and temperature dependence (Can ESM, Zahariev et al., 2008) and phosphate and iron limitation (IPSL-PISCES, Aumont et al., 2015). These methods implicitly assume a constant standing stock of diazotrophs and thereby require fewer assumptions on not well-known ecophysiological processes and parameters compared to models that explicitly model the dynamics of diazotrophs. Explicit parameterizations of N_2_ fixation are mostly based on the assumption that diazotrophs are slow-growing photosynthetic organisms that can fix N_2_. This simple mechanistic approach results in diazotrophs being outcompeted by the faster growing ordinary phytoplankton in N-replete regions, yielding a control by excess PO_4_, or P^∗^, in early on simple box-models (Tyrrell, 1999). Controls by other environmental factors such as temperature, light, iron (CESM, Moore et al., 2013) and also specific trade-offs (e.g., DOP-uptake, Landolfi et al., 2015) or nitrate and oxygen inhibition (GFDL-TOPAZ, Dunne et al., 2013) have been implemented in more complex functional-types ecosystem-ocean circulation models, modulating the rates and regional patterns of fixation. Some studies also parameterize different groups of diazotrophs, in particular Trichodesmium-type, unicellular cyanobacteria, and diatom-cyanobacterial associations (Monteiro et al., 2010). There is a significant spread in global and regional N_2_ fixation estimates by ESMs (**Table 1**). This arises from differences in model parameterizations and parameter values, and the different underlying circulation fields. In restoring approaches, N_2_ fixation is strongly associated with regions of NO_3_ deficits, or high P^∗^, from denitrification in low-oxygen waters (Ilyina et al., 2013). This tight coupling is relaxed in parameterizations with temperature-dependent and Fe-limited growth rates, and also with NO_3_ uptake by diazotrophs (Moore et al., 2013; Landolfi et al., 2017) or NO_3_ inhibition of N_2_ fixation (Zahariev et al., 2008; Dunne et al., 2013; Aumont et al., 2015), which all tend to reduce the simulated rates of N_2_ fixation in NO_3_-replete regions (e.g., Eastern Tropical South Pacific upwelling) and enhance them toward warm, dusty and P-rich waters in line with the traditional paradigm (Redfield et al., 1963; Falkowski, 1997). Most models do not reproduce the observed high fixation rates in the oligotrophic, dusty, North Atlantic due to the modeled low supply of P^∗^ in this region. This implies that unaccounted factors may also be important in controlling marine diazotrophs and require to go beyond the traditional paradigm. In a recent model study, we found that preferential phosphorus remineralization, dissolved organic phosphors uptake (DOP), or Fe-limited growth, were all not able to significantly enhance P^∗^ supply and expand the diazotrophs’ niche in this region. Only accounting for the elevated N-cost of dissolved organic P scavenging allowed the success of diazotrophs in the N-rich P-poor North Atlantic (Landolfi et al., 2015; **Figure 1C**). This theory, however, awaits experimental testing. Current global models do not account for non-cyanobacterial N_2_ fixation, which requires a more in-depth understanding of the relevant players, their ecophysiology and their environmental controls.

## Implications for the N Budget

As N_2_ fixation and denitrification may be uncoupled, the oceanic N inventory is susceptible to imbalances. Despite the relatively short residence time of oceanic N of less than 3000 years (e.g., Somes et al., 2013), the geological isotopic record suggests a close-to-balanced budget over the past 3000 years (Altabet, 2007). This indicates the existence of negative feedbacks that stabilize the marine N reservoir. The nature, intensities and timescales of these feedbacks are still debated. Their effect on time scales shorter than those associated with the global ocean overturning circulation is assumed to require a spatial proximity of regions of N_2_ fixation and N removal, such that any N deficit generated by denitrification in ODZs is rapidly compensated for by N_2_ fixation (Deutsch et al., 2007). However, because denitrification consumes more N than is fixed per mole of organic P, a too tight spatial coupling would lead to net N removal (Landolfi et al., 2013). Thus any N deficit may need to be transported and compensated for far away from ODZ-influenced regions, suggesting that longer (ocean-circulation) timescales may be required to counteract any N imbalance (Landolfi et al., 2013; Somes et al., 2016).

Some studies suggested that N loss by denitrification may exceed, by more than 100 Tg N y^−1^, the inputs of N from N_2_ fixation, atmospheric and riverine supply, as a result of anthropogenic perturbations (Codispoti et al., 2001; Codispoti, 2007). While the recent revised estimates of N_2_ fixation (**Table 1**), atmospheric and river N supply (39 and 34 Tg N y^−1^, respectively, Jickellset al., 2017), and denitrification (120–240 Tg N y^−1^, DeVries et al., 2013) may suggest a reduced imbalance to less than 100 Tg N y^−1^, the annual rate and distribution at which N enters the ocean is still highly uncertain, making current and future N inventory projections speculative. The persistence of a 50–100 Tg N y^−1^ imbalance for 100 years would cause the N inventory to decline by 0.75–1.5% (N inventory of 6.6 10^5^ Tg N, Gruber and Galloway, 2008) possibly weakening the biological carbon pump.

Quantification of non-cyanobacterial N_2_ fixation is currently hampered by the lack of reliable data (Moisander et al., 2017; Benavides et al., 2018) and knowledge of their metabolisms and environmental controls (Riemann et al., 2010; Bombar et al., 2016). However, biogeochemical observations can provide strong constraints on the potential magnitude of this process, which should leave its imprint on the spatial and vertical cumulative distributions of nutrients and the δ^15^NO_3_ signature in the ocean. Although thermodynamically favorable (ΔG < 0), the reduction of N_2_ to NH_3_ requires a large input of energy, from light or organic matter degradation, to proceed at measurable rates (Postgate, 1982) and maintain a low-oxygen cellular environment for the functioning of the enzyme complex nitrogenase. In ODZs the energy requirements may be reduced and iron limitation, a further constraint in oxic surface waters, may be relaxed. Here fixing N_2_ might be energetically advantageous compared with NO_3_-uptake (Großkopf and LaRoche, 2012). However, the low energetic efficiency of the anaerobic metabolism may quantitatively constrain the rates of non-cyanobacterial N_2_ fixation, which may result in invisible (“cryptic”) nutrient and isotopic tracer-distributions patterns as denitrification and anammox may override its signature. Further investigations should asses the contribution of this process.

Our current understanding of stabilizing N-inventory feedbacks stands on the regulatory-competition between N_2_ fixers and non-fixing phytoplankton (Redfield, 1963; Gruber, 2004). N_2_ fixers fertilize the ocean with N until fixed N levels are, relative to P, high enough for their competitive exclusion. The potential for N inputs via non-cyanobacterial N_2_ fixation independent of N deficits would fail this regulatory mechanism. Overall, the detailed mechanisms by which N_2_ fixation responds to N losses are not well understood to have high confidence of marine nutrient cycles future predictions. In this context, the extent to which the North Atlantic stands out as a region of high N_2_ fixation may be a pivotal question. Answering it will help to better understand how tight the feedback is between N_2_ fixation and N loss processes.

## Synthesis and Outlook

All approaches to constrain global rates of N_2_ fixation (experimental, geochemical, and model-based estimates) have their own uncertainties and biases. Albeit the recent progress we still lack a comprehensive knowledge of the relevant environmental and ecological interactions, which prevent us from making robust N_2_ fixation rates estimates and predictions. To reduce the current level of uncertainty an improved understanding of the players, their ecological interactions, and the factors that control N_2_ fixation in the ocean must be drawn from a collective research effort. This requires to take novel approaches that go beyond assuming simple correlations/sensitivities, but rather consider a combination of environmental factors and emerging ecological interactions, both in the field and in the laboratory. To this end, identifying essential traits and ecological interactions experimentally, and develop mechanistic based models, may help us decipher the complexity of marine N_2_ fixation and allow robust fixation estimates. As of now, the large uncertainties in existing N_2_ fixation estimates and in the underlying feedbacks with N loss processes rule out reliable predictions. This task needs a fresh turn.

## Author Contributions

AL designed and wrote the paper. All authors contributed to the writing of the paper.

## Conflict of Interest Statement

The authors declare that the research was conducted in the absence of any commercial or financial relationships that could be construed as a potential conflict of interest.

## Footnotes

1 https://www.us-ocb.org/n-fixation-working-group/
